# Parent-Led Applied Behavior Analysis to Impact Clinical Outcomes for Individuals on the Autism Spectrum: Retrospective Chart Review

**DOI:** 10.2196/62878

**Published:** 2024-10-30

**Authors:** Anurag Garikipati, Madalina Ciobanu, Navan Preet Singh, Gina Barnes, Frank A Dinenno, Jennifer Geisel, Qingqing Mao, Ritankar Das

**Affiliations:** 1 Montera, Inc. dba Forta, 548 Market St, PMB 89605, San Francisco, CA, 94104-5401, United States, 1 415 322 8857

**Keywords:** applied behavior analysis, autism spectrum disorder, parent training, patient outcomes, skill acquisition, pediatrics

## Abstract

**Background:**

Autism spectrum disorder (ASD) can have traits that impact multiple domains of functioning and quality of life, which can persevere throughout life. To mitigate the impact of ASD on the long-term trajectory of an individual’s life, it is imperative to seek early and adequate treatment via scientifically validated approaches, of which applied behavior analysis (ABA) is the gold standard. ABA treatment must be delivered via a behavior technician with oversight from a board-certified behavior analyst. However, shortages in certified ABA therapists create treatment access barriers for individuals on the autism spectrum. Increased ASD prevalence demands innovations for treatment delivery. Parent-led treatment models for neurodevelopmental conditions are effective yet underutilized and may be used to fill this care gap.

**Objective:**

This study reports findings from a retrospective chart review of clinical outcomes for children that received parent-led ABA treatment and intends to examine the sustained impact that modifications to ABA delivery have had on a subset of patients of Montera, Inc. dba Forta (“Forta”), as measured by progress toward skill acquisition within multiple focus areas (FAs).

**Methods:**

Parents received ≥40 hours of training in ABA prior to initiating treatment, and patients were prescribed focused (≤25 hours/week) or comprehensive (>25‐40 hours/week) treatment plans. Retrospective data were evaluated over ≥90 days for 30 patients. The clinical outcomes of patients were additionally assessed by age (2-5 years, 6-12 years, 13‐22 years) and utilization of prescribed treatment. Treatment encompassed skill acquisition goals; to facilitate data collection consistency, successful attempts were logged within a software application built in-house.

**Results:**

Improved goal achievement success between weeks 1‐20 was observed for older age, all utilization, and both treatment plan type cohorts. Success rates increased over time for most FAs, with the exception of executive functioning in the youngest cohort and comprehensive plan cohort. Goal achievement experienced peaks and declines from week to week, as expected for ABA treatment; however, overall trends indicated increased skill acquisition success rates. Of 40 unique combinations of analysis cohorts and FAs, 20 showed statistically significant positive linear relationships (*P*<.05). Statistically significant positive linear relationships were observed in the high utilization cohort (communication with *P*=.04, social skills with *P*=.02); in the fair and full utilization cohorts (overall success with *P*=.03 for the fair utilization cohort and *P*=.001 for the full utilization cohort, and success in emotional regulation with *P*<.001 for the fair utilization cohort and *P*<.001 for the full utilization cohort); and in the comprehensive treatment cohort (communication with *P*=.001, emotional regulation with *P*=.045).

**Conclusions:**

Parent-led ABA can lead to goal achievement and improved clinical outcomes and may be a viable solution to overcome treatment access barriers that delay initiation or continuation of care.

## Introduction

Autism spectrum disorder (ASD) impacts multiple domains of functioning and quality of life, which can persevere through an individual’s lifetime [1,2]. These impacts span an individual’s interpersonal relationships; intrapersonal experience and well-being; family finances; and parental stress [3-5]. Mitigating the impact of ASD on the long-term trajectory of an individual’s life and the immediate familial well-being requires early and adequate treatment. Applied behavior analysis (ABA), a gold-standard treatment for ASD, employs scientifically validated and evidence-based approaches to foster skill acquisition across varying domains [6-8]. ABA is typically prescribed within the patient’s treatment plan as comprehensive (treatment intensity ~25‐40 hours/week) or focused (treatment intensity ~10‐25 hours/week) treatment [9,10], as determined by a board-certified behavior analyst (BCBA). ABA treatment can be delivered via a behavior technician (BT) meeting age, education, and training requirements (>40 training hours) with BCBA oversight [9].

Although treatment success for ABA has extensive documentation in literature, this treatment approach suffers from several challenges that impede access and implementation [6-8]. Shortages in the BT and BCBA workforce result in difficulties with access and wait lists, particularly for individuals residing in remote geographical areas, leading to treatment delays [11-13]. Although the Behavior Analyst Certification Board (BACB) provides general guidelines, the traditional ABA model lacks a standard approach, as treatment plans need to be individualized. With the prevalence of ASD growing continually over the past several decades, expanding ABA access is necessary to ensure that individuals on the autism spectrum can develop and master social, emotional, and daily living skills [13].

The proximity of parents and/or caregivers (hereinafter referred to as “parents”) to individuals on the autism spectrum within daily living environments presents an opportunity to supplement or deliver naturalistic ABA treatment. In fact, many health insurance companies require a parent component (eg, participation in treatment) in order to ensure that treatment progress can be generalized in different settings [14]. Parent-led therapies have the added benefit of eliminating barriers such as wait lists and scheduling difficulties that occur with ABA treatment in clinical settings. Research has validated the effectiveness of parent-led therapies/interventions for ASD and other neurodevelopmental disorders [11,15-21]. Heitzman-Powell et al used a virtual platform to train parents in geographically remote areas to deliver ABA, after which parents gained an average of >39% increase in ABA knowledge; their successful ABA implementation also increased by >40% [11]. Molnár et al examined a parent-delivered early intensive behavioral intervention (EIBI), a form of ABA, and observed improvements in outcomes across different metrics [22]. Oono et al conducted a systematic review of trials examining parent-led interventions for children on the autism spectrum, and they found statistically significant improvements in language/communication and a reduction in ASD severity [23].

Our study contributes to the body of research on patient outcomes resulting from parent-led ABA. However, our work stands out from most prior work on parent- or caregiver-delivered ABA, as we present the outcomes of sustained real-world treatment outside of a research setting, which has the downside of a defined and relatively short study period. Our previous pilot study [18], which is related to this work, examined how nontraditional ABA modalities benefitted patient outcomes compared to traditional ABA. In this work, we report upon the sustained impact that modifications to ABA delivery have had on a subset of patients of Montera, Inc. dba Forta (hereinafter, “Forta”). Particularly, we describe how individuals receiving parent-led ABA treatment progressed toward goal achievement in terms of skill acquisition within multiple focus areas (FAs). Notably, this study is not a research trial study, but rather a retrospective chart review reporting on clinical outcomes of patients in parent-led ABA treatment.

## Methods

### Overview

**Figure 1.**
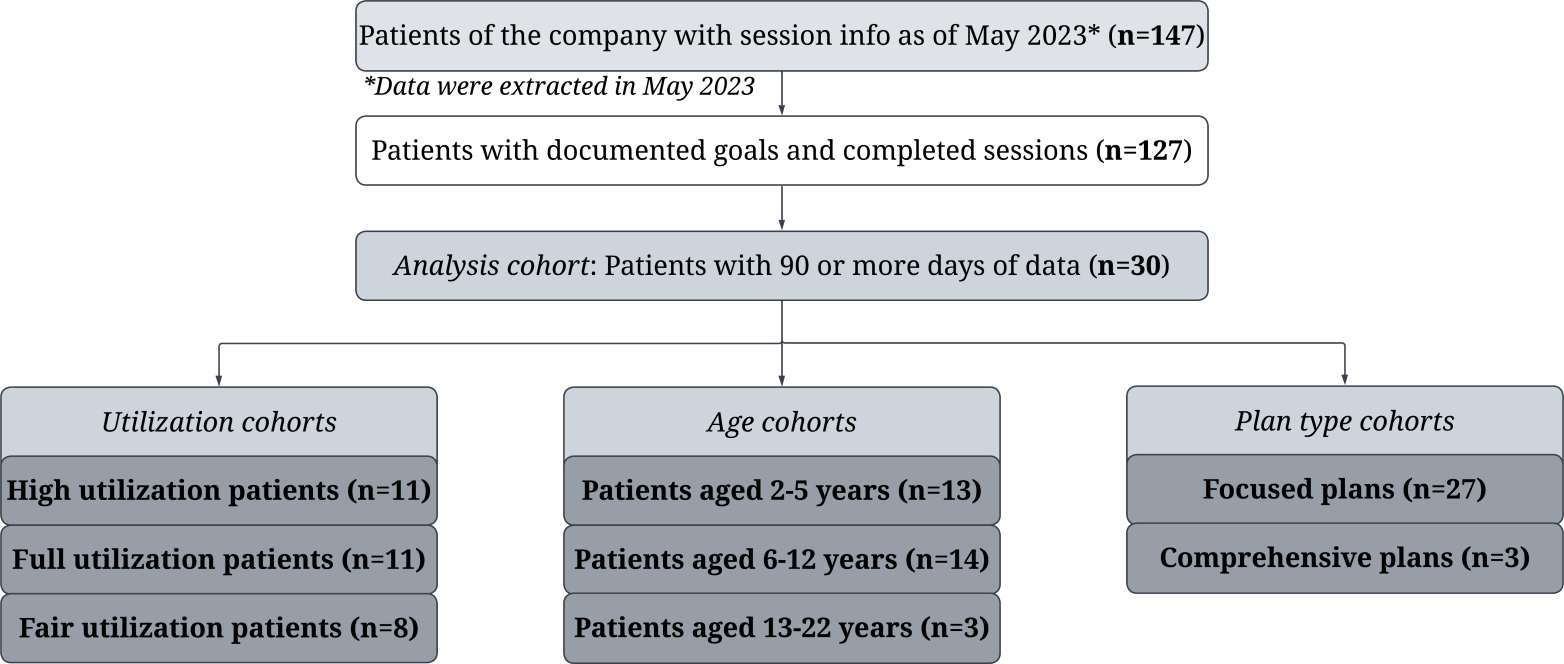
Attrition chart. Figure created using Lucidchart [38].

Active patients of Forta between October 2022-May 2023 with a diagnosis of ASD per the Diagnostic and Statistical Manual of Mental Disorders, Fifth Edition (DSM-5) diagnostic criteria [24] were considered for the study. Patients with incomplete documentation of goal progress and without a minimum of 90 days of data were excluded. The 90-day threshold ensured patients would be at least halfway through a typical 6-month ABA treatment plan. This resulted in 30 patients with sufficient data for analysis, as shown in the attrition chart (Figure 1). Patients included in the data analysis had available longitudinal data ranging from 15‐20 weeks. Variation in available data resulted from patients initiating treatment with the company at different time points. Prior to treatment delivery, parents of patients completed ≥40 hours of ABA training using a virtual, web-based program adhering to BACB standards [25]. After training, the parents were required to pass an Initial Competency Assessment to demonstrate the skills and knowledge required to deliver ABA treatment. Upon successfully passing the Initial Competency Assessment, the parents became BTs and were assigned to a BCBA for supervision of the treatment process in accordance with the BACB guidelines [25]. Unlike typical BTs that deliver ABA treatment to more than one patient during a given week, the parents that became BTs focused on leading treatment delivery solely for their own children.

Demographics for the 30 patients whose data were processed and analyzed are displayed in Table S1 in Multimedia Appendix 1. Demographic data were obtained from ABA treatment patient intake forms completed by parents. The average patient age was 8.39 years (aged 2.81‐22.60 years at the start of their data collection), and 77% (23/30) of the patients were males. Within the total patient cohort, 2 patients had syndromic ASD (both with DiGeorge syndrome, mild ASD, took no medication) and 28 patients had nonsyndromic ASD, where syndromic versus nonsyndromic ASD was defined according to Genovese and Butler [26]. At the beginning of the study, 14 patients took no medication, 3 were administered nonprescription medication or supplements (eg, antihistamines, multivitamins, probiotics, melatonin), 5 were administered antipsychotics, and 8 were administered prescription medication other than antipsychotics (eg, stimulants for attention-deficit/hyperactivity disorder, replacement hormones, anticonvulsants). We are not aware of any medication changes during the course of the study.

Patients were grouped by utilization of the number of ABA treatment hours that were prescribed by the BCBA who conducted the intake assessment and provided treatment oversight (Figure 1). To determine each utilization cohort, utilization data for each patient were averaged over their individual studied time period (≥15 weeks) and that average was used to classify each patient as having fair, full, or high utilization. The high utilization patient cohort completed ≥95% of the prescribed ABA treatment hours; the full utilization patient cohort completed 80% to <95%; and the fair utilization patient cohort completed <80%. Patients were assessed within three age cohorts (2.00‐5.99 years, 6.00‐12.99 years, and 13.00‐22.99 years) and by their assignment to a focused (<25 hours/week) or comprehensive (>25 to 40 hours/week) ABA plan (Figure 1). The age cohorts align with existing research examining effects of ABA treatment across various ages [27].

**Figure 2.**
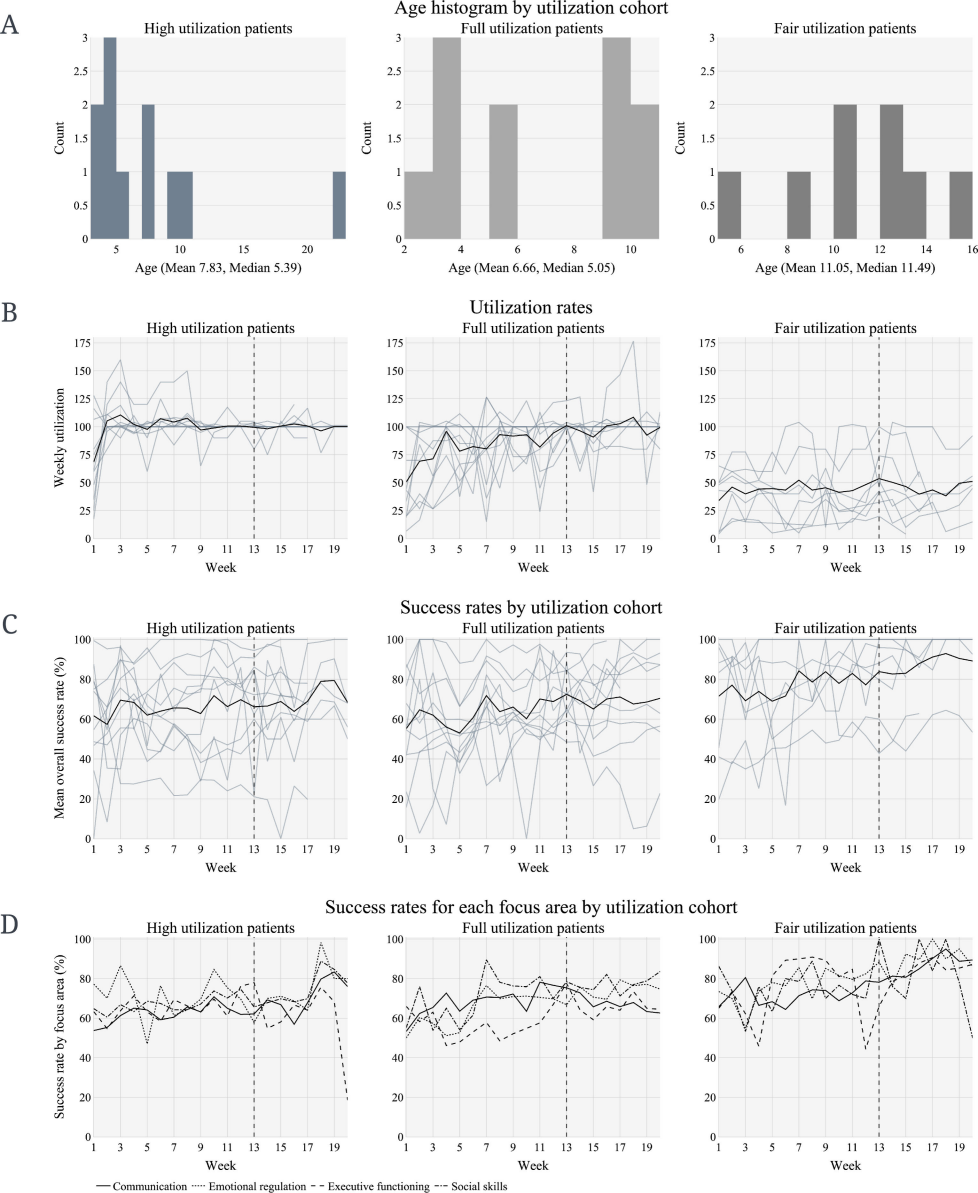
High utilization cohorts by (**A**) age and (**B**) weekly utilization. Goal achievement/success rate by utilization (**C**) overall and (**D**) for each focus area. Black lines aggregate all patients; each gray line represents a single patient; a vertical dashed line at 13 weeks denotes the cutoff for the minimum amount of data that each patient was required to have for inclusion in the study. This figure was created using Plotly in Python.

The patient age distribution in each utilization cohort is shown in Figure 2A. There were 11 patients in the high utilization cohort (received ≥95% of prescribed treatment), 11 patients in the full utilization cohort (received 80% to <95% of prescribed treatment), and 8 patients in the fair utilization cohort (received <80% of prescribed treatment). Figure 2B displays overall utilization rate trends for all patients within each utilization cohort. Weekly utilization was averaged on a weekly basis for each patient and represents the percentage of prescribed weekly ABA treatment hours that patients utilized during the study. Previous studies indicated that utilization of >80% of prescribed ABA hours can be regarded as a “full dose” of ABA treatment (ie, full utilization) [28,29].

Following the intake assessment, the BCBA provided each patient with a highly individualized treatment plan establishing specific goals for skill acquisition based on that individual patient’s strengths and weaknesses across multiple FAs. For complex skill acquisition goals, goals were broken down into multiple treatment targets for the patient to master the entire goal. For example, within the communication domain, if a patient was prescribed the skill acquisition goal of “identifying common objects,” the patient could be further assigned the targets of “identifying a chair” and “identifying a cup” to work on. The process of mastering each goal involved the patient working on one or more targets associated with that specific skill acquisition goal. On average, patients in the analysis cohort worked on 21.1 skill acquisition goals and associated targets during the study period.

To measure clinical outcomes for each patient, the study solely evaluated skill acquisition goals. Patient progress toward mastering a particular skill was assessed with quantifiable parameters (ie, number of successful attempts to complete a task). This evaluation served as an indicator of the patient’s level of achievement toward a specific goal, a measurement technique that may be better suited to indicate clinical progress and outcomes than standard of care assessments [30]. The goal achievement data are reported as the percentage of successful attempts to complete a task out of all attempts, reported as percent of trial data. The goal achievement data were averaged weekly for each patient for each skill acquisition goal they worked on during that week. Goal achievement data are expressed as a percentage value ranging from 0% (0 successful attempts) to 100% (all attempts were successful). This is a method similar to that used by Choi et al, in which patient progress in ABA treatment was measured by the desired percent of goals versus actual goals achieved [31].

Baseline data for each skill acquisition goal for each patient were collected either by BCBA or parent assessment. This provided the opportunity for the BCBA to personalize the treatment plan and progress measurement. Longitudinal goal achievement data for each patient were logged for each skill (subsequent to baseline data measurement for that particular skill) by the parent BT during the course of ABA treatment sessions on a software application built in-house. This application facilitated streamlined data collection and analysis. Following the creation and implementation of a treatment plan, specific goals were tracked on the application, which provided a user-friendly interface for parent BTs to log session data, while having the additional benefit of ensuring a robust and streamlined data collection and analysis process. During a treatment session, parent BTs could select a specific skill acquisition goal or target thereof from the assigned treatment plan and report the total number of attempts and how many were successful. As data were easily accessible, parent BTs could review data after the treatment session to complete notes and progress reports. The application also provided a user-friendly mechanism for the BCBAs to track progress over time and ensure the most pertinent skill acquisition goals were being implemented and evaluated. Data for the analysis in this study were collected through the application, encompassing the data for all skill acquisition goals across all treatment sessions for the 30 patients with ≥90 days of data. With direct clinical data, there is a limitation on interrater reliability, as each patient was only evaluated by a single practitioner providing therapy (ie, their parent BT). However, our treatment delivery methods were evidence-based (eg, skill acquisition via manding [a request for a want or need], reinforcement, task analysis) and BCBAs performed ongoing supervision sessions with parent BTs and patients, thereby monitoring overall progress to ensure treatment was administered effectively and within the ABA standard of care [9]. Parent BTs performing ABA treatment were supervised by a BCBA for at least 5% of the treatment time (according to the BACB requirement) [25], or for any amount of treatment time required by state or insurance regulations [32-36].

For data analysis, we grouped skill acquisition goals into 4 FAs or categories: communication (COM), emotional regulation (ER), executive functioning (EF), and social skills (SS). Each FA aligned with a corresponding domain or subdomain utilized in the Vineland Adaptive Behavior Scale, 3rd Edition (Vineland-3), a widely used assessment tool targeting neurodevelopmental disorders, as follows [37]. COM FA corresponds to the Vineland COM domain; ER FA to the Coping Skills subdomain (Socializing domain); EF FA to the Daily Living Skills domain; and SS FA to the Vineland Interpersonal Relationships and Play and Leisure subdomains (Socializing domain). Vineland-3 domains and subdomains were used strictly for mapping the FAs (ie, assigning every skill acquisition goal to an FA) and were not employed as a data assessment scale for the data reported in this paper.

To evaluate the progress of patients and compare different cohorts, patients were first grouped into utilization rate, age groups, and treatment plan type cohorts. Within each group, goal success rate was measured across all skill acquisition goals and across each FA across cohorts (eg, success rate of all ER goals across all patients with a comprehensive care plan) for each patient. To evaluate the progress of goals over time, a correlation coefficient was computed for each FA for each cohort to determine the magnitude of change and the direction of the success rate trend for each cohort. A statistical significance test was then performed across all patient cohorts to determine if the correlation coefficient (*r*) was significant, indicating the change in performance over time did increase over the evaluation period of 16 weeks (Table 1).

**Table 1.** Summary table indicating the net change in success rate overall and across each focus area for the different utilization, treatment plan type, and age cohorts (weeks 1‐16).^a^

		Utilization cohorts			Treatment intensity cohorts		Age cohorts (years)		
		High (n=11)	Full (n=11)	Fair (n=8)	Focused (n=27)	Comprehensive (n=3)	2‐5 (n=13)	6‐12 (n=14)	13‐22 (n=3)
**Autism spectrum disorder severity breakdown**									
	Mild	2	5	4	10	1	2	7	2
	Moderate	4	4	2	9	1	7	3	0
	Severe	5	2	2	8	1	4	4	1
**Age (years), mean (SD)**		8.2 (5.4)	6.7 (3.3)	11.0 (3.0)	8.9 (4.3)	4.0 (1.0)	4.3 (1.0)	10.1 (1.4)	17.0 (4.9)
Utilization percentage, mean (SD)		100.1 (3.4)	88.1 (4.6)	42.1 (24.3)	78.8 (28)	92.8 (8.8)	88.7 (23.1)	78.5 (24.5)	51.6 (41.7)
Prescribed hours, mean (SD)		21.8 (3.4)	21.4 (5.5)	23.1 (2.6)	21.1 (3.2)	30.0 (0.0)	23.5 (4.3)	20.7 (3.7)	21.7 (2.9)
Number of goals and associated targets, mean (SD)		18.5 (7.7)	21.5 (10.2)	24.0 (12.5)	21.0 (9.7)	21.7 (14.6)	18.5 (9.8)	24.6 (9.9)	16.3 (8.1)
**Medication breakdown**									
	None	7	3	4	13	1	6	7	1
	Meds^1b^	0	2	1	3	0	2	0	1
	Meds^2c^	1	3	1	4	1	2	3	0
	Meds^3d^	3	3	2	7	1	3	4	1
**Communication**									
	*r*	0.500	0.450	0.748	0.645	0.719	0.455	0.616	0.153
*P* value	.04	.07	.001	.05	.001	.07	.008	.62
**Emotional regulation**									
	*r*	−0.200	0.772	0.773	0.737	0.492	0.677	0.549	0.275
	*P* value	.44	<.001	<.001	.001	.045	.003	.03	.30
**Executive functioning**									
	*r*	0.136	0.410	0.305	0.544	0.382	0.224	0.536	−0.237
	*P* value	.60	.10	.23	.02	.13	.39	.03	.36
**Social skills**									
	*r*	0.570	0.537	0.370	0.664	0.466	0.510	0.516	0.592
	*P* value	.02	.03	.14	.004	.06	.04	.03	.09
**Overall**									
	*r*	0.020	0.798	0.597	0.646	0.358	0.319	0.330	0.360
*P* value	.95	.001	.03	.02	.23	.29	.27	.23

a *P*<.05 indicates significant likelihood of data having a linear relationship.

b Meds1: nonprescription medication.

c Meds2: prescription medication, antipsychotics.

d Meds3: prescription medication, other than antipsychotics.

### Ethical Considerations

This work was deemed exempt by an independent Institutional Review Board per Food and Drug Administration 21CFR56.104 and 45CFR46.104(b)(4) and received a waiver of informed consent. The work was carried out in accordance with ethical standards and with the Declaration of Helsinki (revised in 2000). Strengthening the Reporting of Observational Studies in Epidemiology (STROBE) reporting guidelines have been applied in this study.

## Results

### Overview

Results are shown for goal achievement progress between weeks 1 (start of data collection) and 20. All patients had available data for the initial 13 weeks of the study and a minimum of 90 days of data total. All selected patients had >15 weeks of data (29 patients with ≥16 weeks, 1 patient with 15 weeks). The data between weeks 16‐20 were less robust, as each patient had a different longitudinal dimension in that time period of data analysis, with some patients having only 16 weeks of data, and others having the full 20 weeks.

### Goal Achievement/Success Rate by Utilization Rate and FA

As shown in Figure 2C, overall success toward skill acquisition increased for all 3 utilization cohorts. Overall success rate appears to be consistent and shows improvement over the 20-week period across all utilization cohorts. When examining the success rate for each FA by utilization cohort for the initial 16-week period, patients in each utilization cohort displayed growth in all 4 FAs, except ER for the high utilization cohort, which varied significantly during this period (Figure 2D). From weeks 16 to 20, patients in the high utilization cohort showed a distinguishable increase in success rate in ER, while remaining highly variable. In addition, patients in the high utilization cohort showed a drop in success for EF from weeks 19 to 20 after a steady increase during the initial 19 weeks. Further, patients in the fair utilization cohort displayed a substantial drop in success rate for SS from weeks 18 to 20, after having increased during the initial 18 weeks. Notably, EF and SS exhibited high variability.

### Goal Achievement/Success Rate by Treatment Intensity (Plan Type) and FA

**Figure 3.**
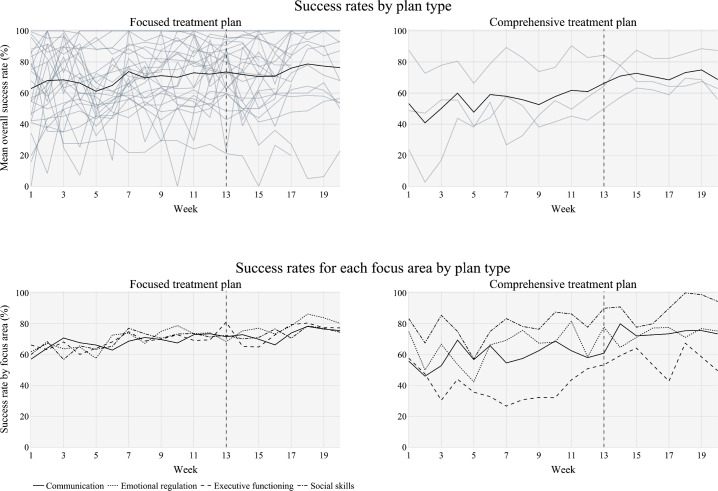
Goal achievement/success rate by treatment intensity: overall (top panels) and for each focus area (bottom panels). Black lines aggregate all patients; each gray line represents a single patient; a vertical dashed line at 13 weeks denotes the cutoff for the minimum amount of data that each patient was required to have for inclusion in the study. This figure was created using Plotly in Python.

For patients in both treatment plan types, growth over time was displayed in the overall success toward skill acquisition (Figure 3, top panels). Success rates for each FA by treatment intensity (Figure 3, bottom panels) followed largely the same trend as the overall success rate (Figure 3, top panels), except EF for the comprehensive treatment plan cohort. Patients prescribed a focused treatment plan displayed consistent and similar growth in success rates over time for all 4 FAs. Patients prescribed a comprehensive treatment plan displayed growth in success rates over time in COM and SS. The success rate for ER and EF showed great variability for the comprehensive treatment plan cohort. However, this trend may be due to a smaller sample size (n=3) compared to the patient cohort prescribed a focused treatment plan (n=27).

### Goal Achievement/Success Rate by Age Cohort and FA

**Figure 4.**
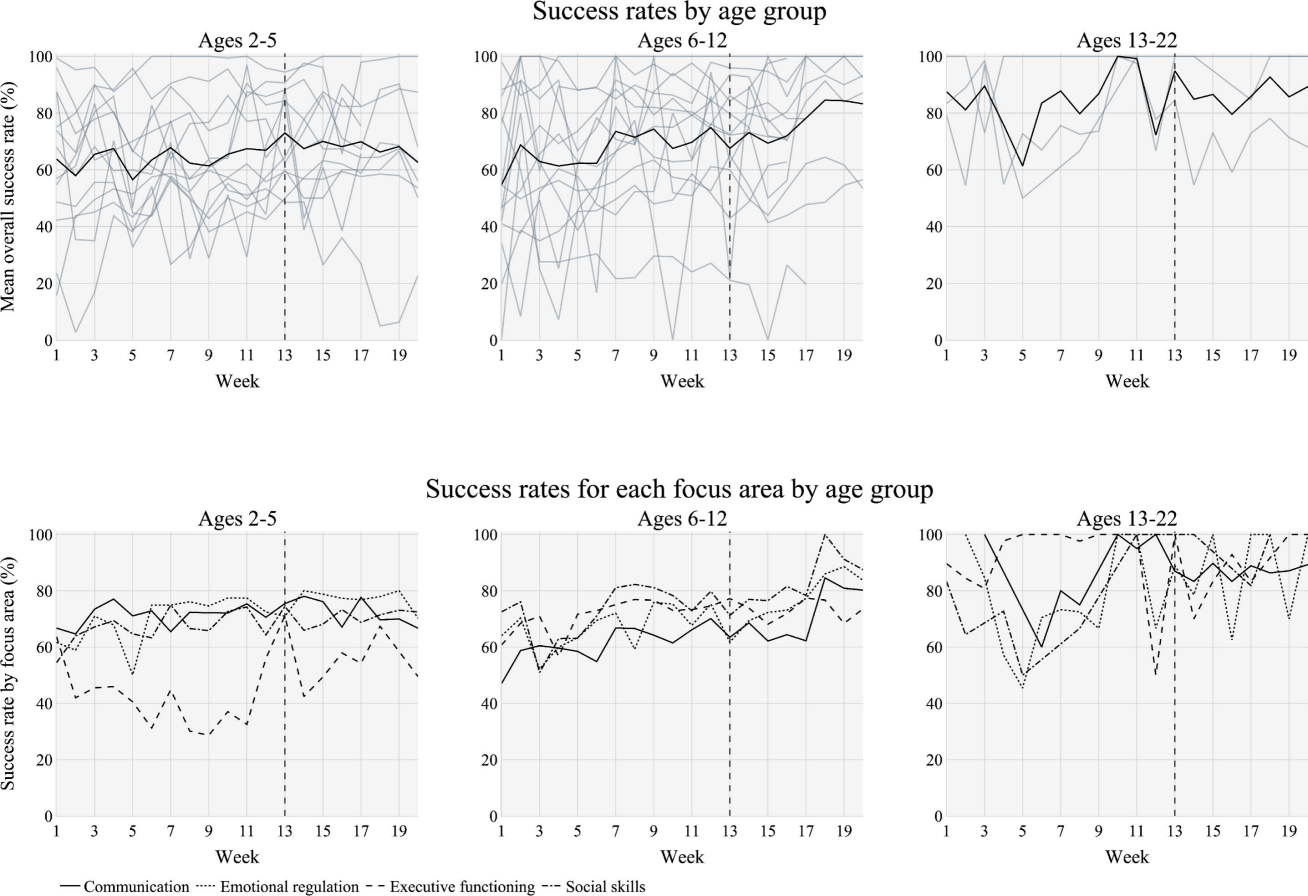
Goal achievement/success rate by age cohort: overall (top panels) and for each focus area by age (bottom panels). Black lines aggregate all patients; each gray line represents a single patient; a vertical dashed line at 13 weeks denotes the cutoff for the minimum amount of data that each patient was required to have for inclusion in the study. This figure was created using Plotly in Python.

As shown in Figure 4 (top panels), overall success toward skill acquisition varied among the 3 age cohorts, with the most notable growth over time for the middle age cohort (6‐12 years), followed by more modest growth for the oldest age cohort (13‐22 years). The youngest age cohort (2‐5 years) displayed growth for the initial 16-week period, where data were present for all but 1 patient. After 16 weeks, less data were available for the youngest and middle age cohorts; thus, statistical significance (as denoted by *P* value and *r*) of the results decreased by comparison with the initial 16-week period. Results after week 16 did not demonstrate statistical significance. The success rates for each FA by age cohort (Figure 4, bottom panels) followed largely the same trend as the overall success rate observed in Figure 4, top panels. The most notable and consistent growth over time was observed for the middle age cohort (6‐12 years) for all 4 FAs. When examining data for the oldest age cohort (13‐22 years) for each FA, no discernible pattern emerged; however, data appear to indicate a trend of increased performance with increasing treatment time. The youngest age cohort (2‐5 years) demonstrated growth for the initial 16-week period for COM, ER, and SS, with ER and SS maintaining growth after 16 weeks. The youngest age cohort struggled the most with EF. It is notable that 3 of the 13 patients in the youngest age cohort have been prescribed a comprehensive treatment plan, and that the EF FA displays the same trend with respect to the other 3 FAs for the comprehensive treatment plan cohort and for the youngest age cohort.

The results in Table 1 indicate the severity breakdown for each analysis cohort, as derived from DSM-5 severity level criteria, and the correlation coefficient (*r*) and *P* value for the cohort’s success rate across each analysis cohort. The correlation coefficient represents the strength of the relationship of the cohort’s success rate over time, with values closer to −1/1 indicating a stronger negative/positive relationship in success rate over time, and values closer to 0 indicating a flatter relationship over time. The corresponding *P* value indicates the likelihood of the relationship being flat (slope of a regression line equal to 0). For each analysis cohort, these values were computed across each FA, as well as overall (ie, in combination across all FAs).

Of the 40 unique combinations of analysis cohorts and FAs (Table 1), 20 showed statistically significant positive linear relationships (*P*<.05), with 26/40 having *r*>0.4, indicating at least a moderate-to-strong positive linear relationship in the data and 35/40 having *r*>0.2, indicating at least a weak-to-moderate positive linear relationship [39].

The mean age of patients between the treatment intensity cohorts indicates that patients with comprehensive treatment plans are younger than patients with focused plans, with all 3 patients in the comprehensive cohort being in the age range of 2‐5 years old. In addition, the youngest age cohort also had the highest mean number of authorized hours, with 3 of the 13 patients prescribed a comprehensive treatment plan. This aligns with literature indicating that younger children will gain more in ABA treatment with more hours [40]. Patients with comprehensive plans also had higher mean utilization, indicating not only a more intensive treatment plan, but also a more intense implementation of the treatment plan. Similarly, younger patients also have substantially higher utilization rates than older patients.

## Discussion

### Principal Findings

This study examines outcomes for a small sample of Forta’s patients receiving parent-led ABA treatment, where half of the cohort combinations demonstrated statistical significance in regard to positive linear relationships for skill acquisition. Within all cohorts, we generally observed growth toward overall success for skill acquisition for all 3 utilization cohorts, with a few exceptions. Additionally, we observed variability in success toward skill acquisition over the study period. Use of success toward skill acquisition as an outcome measure fills a gap in the literature, as a majority of studies utilize standardized measurements, such as Vineland-3, which do not reflect the granular changes that can be observed with behavior changes [8,27]. This is of particular value in clinical settings, as these changes can guide individualized treatment planning.

Separation of patients by age (cohort 1: 2‐5 years, preschool; cohort 2: 6‐12 years, elementary and middle school; cohort 3: 13‐22 years, high school and older) reflects research indicating that these 3 age groupings master different skills at different rates within ABA treatment. This is particularly true for younger children, for whom the number of treatment hours directly impacts success toward skill acquisition [27].

When stratified by utilization, all 3 cohorts generally experienced growth in skill acquisition. The variability in success with ER within the high utilization cohort could be the result of parents having to spend a significant amount of time getting patients to become receptive to treatment during a session (owing to patient struggles with ER and EF).

Within cohorts stratified by age, there were several statistically significant positive linear relationships observed for the middle age cohort in all FAs, which may indicate that these patients were more receptive to treatment. The youngest age cohort struggled with EF; however, attaining EF skills is known to be a complex process that may be impacted by subtle differences between individual patients [41]. This cohort was the only age cohort for whom progress in EF goals declined between the start and conclusion of the study, which could be attributed to a number of factors. For example, this age cohort had the most patients with a high level of symptom severity, which may have impeded progress toward EF skill acquisition. Progress may also have been hindered if parents had to spend a greater number of hours acclimating their children to the treatment process.

Regarding the variability from week to week for skill acquisition success, research indicates that progress toward skill acquisition in ABA treatment does not typically follow a steady progression; thus, variability is expected [8,19,37,40]. Observing such fluctuations within different FAs may help a parent decide which skill to focus on for a given day, depending on their child’s success level [27]. Although we also detected this weekly variability, several FAs followed linear trends in terms of progress within each cohort. Regarding the linearity that was observed within the focused treatment cohort across all FAs, this demonstrated that fewer treatment hours (focused treatment plans have fewer hours than comprehensive ones) can also lead to linear skill gains. Our results indicate that fewer treatment hours can also be beneficial; however, according to the existing literature, a higher number of hours (ie, comprehensive treatment) provides for better outcomes in terms of skill acquisition in ABA for individuals on the autism spectrum [42]. Linstead et al noted that when treatment intensity was either high or low, individuals did not gain as much per hour as individuals who received an intermediate number of treatment hours [30]. These two literature findings [30,42] are not mutually exclusive and both support the notion that treatment plans have to be highly individualized to provide the basis for achieving the most beneficial treatment gains. The linearity of gains for the patients in the focused cohort may indicate that the focused plan was appropriately assigned for the 27 patients. Further, even with only 3 patients in the comprehensive cohort (not providing much opportunity for averaging out data outliers), COM and ER displayed statistically significant positive linear relationships (*P*<.05, Table 1), which may also indicate that the focused treatment plan was appropriately assigned for the 27 patients.

When the patients were stratified by treatment intensity (plan type), there was more variation in the level of skills within all focus areas at both the start and conclusion of the study in the comprehensive versus focused cohorts.

Data regarding broad average utilization based on either prescribed hours of treatment or insurance-approved hours of treatment are not publicly documented, to the best of our knowledge. Therefore, the utilization rates that we present in this clinical outcomes study will fill a gap in the literature. Within research studies of ABA treatment, rates of utilization are highly variable. However, variability in the use of prescribed ABA hours and underutilization of prescribed ABA hours, which may impact a patient’s ability to receive the “full dose” of treatment (at least 80% of prescribed treatment hours), is not uncommon. In a study by Yingling et al, patients on the autism spectrum who were prescribed a specific number of weekly hours for EIBI treatment only used a mean of 37% of those hours and the authors further noted that the most utilization was observed within the first week of EIBI treatment [43], a pattern that was not observed within our patient cohorts. By contrast, our overall utilization in the analysis cohort (n=30) was 80.2%, which is over twice as high when compared with the utilization reported by Yingling et al [43]. Choi et al examined ABA treatment utilization and noted that 28% of the patient population received the “full dose” of treatment in terms of hours or sessions [44], in contrast to our study, in which 70% of the patients used the “full dose” of authorized treatment hours. Croen et al [29] studied an ABA-based behavioral health treatment in which underutilization of prescribed treatment hours was observed, with only 15% of patients receiving the “full dose” of prescribed treatment hours. These studies examined treatment in clinical settings (as opposed to treatment delivered in-home by parents), and there may have been barriers that were prohibitive for initiating and maintaining care, such as a nationwide shortage of ABA treatment providers [45] or geographical limitations and/or costs associated with travel to obtain treatment in clinical settings, which are mitigated in our treatment approach [11]. Differences between the high utilization of the prescribed hours in our study and relatively poor treatment adherence in other studies could also be attributed to the parents’ increased confidence in their ability to successfully deliver ABA treatment resulting from the receipt of ABA training [21,46].

This research highlights the potential value of parent-led ABA treatment in terms of impacting a patient’s success toward goal acquisition, particularly as it relates to overcoming financial and logistical burdens of gaining access to ABA [16]. These factors impede the ability to obtain and sustain treatment, which prevents patients on the autism spectrum from receiving validated treatment that may improve outcomes [15,45]. At the time the data were analyzed for this study, Forta had almost 200 patients, a number which has seen continued and substantial growth in a small window of time. This may reflect the need for more service providers, as well as the need for alternative ABA treatment delivery methods that afford greater flexibility (ie, in-home and parent-led) for parents and families, which may ensure treatment uptake and adherence. By training parents to deliver treatment to their children on their own schedule and without having to leave their homes, parents can continue treatment regardless of the availability of BTs to sustain the gains that their child makes toward skill acquisition. Additionally, by allowing the BCBAs to perform monitoring virtually, logistical barriers are eliminated and the BCBAs can use time that may otherwise have been used for travel to patients’ homes to see more patients. Parent-led ABA further allows treatment to be delivered in a culturally appropriate manner, which is important for treatment outcomes [16,47].

Study limitations are as follows. First, our study did not account for variables beyond age, utilization, and treatment intensity. Research suggests that sociodemographic traits can impact the utilization of ABA, which may impact progress toward goal achievement [48]. The severity of symptoms and the parent’s skills for treatment delivery may also impact treatment outcomes [42]. In our 2 youngest cohorts, a higher number of patients were diagnosed with moderate and severe ASD. Though these cohorts accessed a “full dose” of treatment (>80% of prescribed hours), the severity of symptoms may require more time in treatment to progress at a pace similar to older patients. Individuals with greater severity of symptoms may also start treatment with limited baseline skills, which may impact rate of progress [49]. Individuals with more severe symptoms may have lower treatment adherence [50], which may impact outcomes. Future work should examine the effect of diverse variables/factors on treatment progression. As parents recorded the progress of their child, there is the potential that response bias may have impacted the internal validity of results. Future examination of patient outcomes may use a more research-focused design, including a control group, randomization of treatment intensity, or a larger number of patients to demonstrate statistical significance, the latter which may also improve upon generalizability. Though our study examined outcomes over a longer period of time than our previous pilot study, observing outcomes over a sustained period of time may elucidate different skill acquisition patterns, as patients are known to demonstrate better achievement in some skills over a longer treatment period (as opposed to intensity) [30]. Measuring such changes may be facilitated by the use of standardized assessments such as Vineland-3. Therefore, future work can incorporate validated outcome measurements to determine larger-scale, longitudinal changes resulting from ABA [8,27]. Last, there was a very small number of patients in the oldest cohort (13‐22 years), which may have prevented the emergence of clearer trends. This cohort also had the lowest utilization, which may be the result of resistance to therapeutic treatments among adolescents (and a greater level of health care decision autonomy), fear of autism-related social stigma [51,52], and/or social and academic obligations. To better understand the extent that outcomes can be impacted in adolescents and young adults, future work should include a greater number of individuals within that age range.

### Conclusion

This retrospective chart review study explored longitudinal trends related to patients’ success toward achieving skill acquisition goals in parent-led ABA therapy within a real-world treatment setting. We examined the clinical outcomes of patients receiving parent-led ABA and noted overall growth in success for most cohorts and FAs. This study demonstrated the potential for parent-led ABA treatment delivery as an alternative approach to traditional ABA delivery in a clinical setting.

## Supplementary material

10.2196/62878 Multimedia Appendix 1 Demographics table showing patient information.

## Abbreviations

ABA: applied behavioral analysis
ASD: autism spectrum disorder
BACB: Behavior Analyst Certification Board
BCBA: board-certified behavior analyst
BT: behavior technician
COM: communication
DSM-5: Diagnostic and Statistical Manual of Mental Disorders, Fifth Edition
EF: executive functioning
EIBI: early intensive behavioral intervention
ER: emotional regulation
FA: focus area
SS: social skills
STROBE: Strengthening the Reporting of Observational Studies in Epidemiology
Vineland-3: Vineland Adaptive Behavior Scale, 3rd Edition
