# Supplementary material for: Parent-Led Applied Behavior Analysis to Impact Clinical Outcomes for Individuals on the Autism Spectrum: Retrospective Chart Review
Source: JMIR Pediatr Parent. 2024 Oct 30;7:e62878. doi: 10.2196/62878 (PMC11540247; doi:10.2196/62878)
Supplement: Multimedia Appendix 1 [file pediatrics-v7-e62878-s001.docx]

**Supplement**

**Supplemental Table 1.** Demographics table showing patient information: sex (assigned at birth), age, schooling, autism spectrum disorder (ASD) severity, comorbidities, and prior therapy.

| **Demographics** | | **Analysis Cohort (N =30)** |
| --- | --- | --- |
| **Age (years)** | 2-5 | 13 (40.0%) |
|  | 6-13 | 14 (50.0%) |
|  | 13-22 | 3 (10.0%) |
| **Sex (assigned at birth)** | Male | 23 (76.7%) |
|  | Female | 7 (23.3%) |
| **ASD Severity Level (DSM-5)** | Mild | 11 (36.7%) |
|  | Moderate | 10 (33.3%) |
|  | Severe | 9 (30.0%) |
| **Schooling** | Home | 9 (30.0%) |
|  | Regular | 5 (16.7%) |
|  | Special Education | 10 (33.3%) |
|  | None | 5 (16.7%) |
|  | Unknown | 1 (3.3%) |
| **Prior/ Concurrent Therapy** | Prior ABA Therapy | 14 (46.7%) |
|  | Speech Therapy | 19 (63.3%) |
|  | Occupational Therapy | 18 (60.0%) |
|  | Physical Therapy | 5 (16.7%) |
| **Comorbidities** | Attention-deficit/hyperactivity disorder (ADHD) | 11 (36.7%) |
|  | Language Disorders | 5 (16.7%) |
|  | Anxiety | 3 (10.0%) |
|  | Seizures | 3 (10.0%) |
|  | Global Developmental Delay (GDD) | 2 (6.7%) |
| **Medication** | N/A | 14 (46.7%) |
|  | non-prescription | 3 (10.0%) |
|  | antipsychotics | 5 (16.7%) |
|  | prescription, other than antipsychotics | 8 (26.7) |
